# Comparing Optical Coherence Tomography Angiography Metrics in Healthy Chinese and Caucasian Adults

**DOI:** 10.3390/jpm14080834

**Published:** 2024-08-06

**Authors:** Inna Bujor, Jacqueline Chua, Bingyao Tan, Raluca Iancu, Ruxandra Pirvulescu, Aida Geamanu, Mihai Bostan, Eduard Toma, Diana Ionescu, Leopold Schmetterer, Alina Popa-Cherecheanu

**Affiliations:** 1 Department of Ophthalmology, Carol Davila University of Medicine and Pharmacy, 050474 Bucharest, Romania; 2 Department of Ophthalmology, Emergency University Hospital, 050098 Bucharest, Romania; 3 Singapore Eye Research Institute, Singapore National Eye Centre, Singapore 169856, Singapore; 4 Ophthalmology and Visual Sciences Academic Clinical Program, Duke-NUS Medical School, National University of Singapore, Singapore 169857, Singapore; 5 SERI-NTU Advanced Ocular Engineering (STANCE), Singapore 639798, Singapore; 6 Clinical Hospital Dr. V. Gomoiu, 022102 Bucharest, Romania; 7 School of Chemistry, Chemical Engineering and Biotechnology, Nanyang Technological University, Singapore 639798, Singapore; 8 Department of Clinical Pharmacology, Medical University Vienna, 1090 Vienna, Austria; 9 Center for Medical Physics and Biomedical Engineering, Medical University Vienna, 1090 Vienna, Austria; 10 Institute of Molecular and Clinical Ophthalmology, 4031 Basel, Switzerland

**Keywords:** optical coherence tomography angiography, retinal perfusion density, superficial and deep capillary plexus, choriocapillaris, foveal avascular zone

## Abstract

Background: The goal of the present study was to identify differences in retinal microvasculature between healthy Caucasians and healthy Asians in order to provide a better understanding of the variability between different ethnic groups. Methods: In this cross-sectional study, 191 healthy Chinese and Caucasian participants were enrolled. They underwent optical coherence tomography angiography (OCTA) scans with Zeiss Cirrus HD-5000 Spectral-Domain with AngioPlex. Linear regression models were used to investigate the association of OCTA metrics with potential risk factors. Results: Whereas participants in both groups are comparable in age and sex, Chinese participants had a longer axial length, higher spherical equivalent, higher intraocular pressure (*p* < 0.001), and a significantly higher perfusion density of large vessels in the superficial capillary plexus (*p* < 0.001). Regarding the foveolar avascular area (FAZ), Chinese participants had a larger superficial FAZ, a wider superficial FAZ perimeter, and a more circular deep FAZ shape (*p* < 0.001). Conclusions: There are significant differences in the retinal vasculature between Caucasian and Asian eyes as measured using OCTA. This needs to be considered when developing normative databases. Whether such findings relate to inter-racial differences in the incidence of retinal vascular disease remains to be shown.

## 1. Introduction

A wider variety of previous studies has indicated that retinal microvasculature may differ between ethnicities. The majority of studies used fundus photography to assess such differences [1,2,3]. Data from several population-based studies performed around the world were used to evaluate microvascular differences between ethnicities. Indeed, ethnic influences on retinal vessel calibers were observed in addition to the environmental factors and genetic determinants [4,5,6]. Retinal pigmentation could, however, be an important source of error in the measurement of retinal vessel caliber based on fundus photographs [5]. In addition, magnification errors related to myopia and differences in eye length need to be considered.

Optical coherence tomography angiography (OCTA) is a non-invasive imaging technique that provides information about retinal and choroidal blood circulation without injecting a contrast agent [7,8,9,10,11]. OCTA is capable of providing high-quality maps representing the three-dimensional vascular structure of the retina achieved by detecting the movement of erythrocytes within blood vessels. Foveal avascular zone (FAZ), superficial retinal capillary plexus (SCP), deep retinal capillary plexus (DCP), and choriocapillaris can be extracted and quantified from these images.

Several studies reported ethnic differences in OCTA parameters. In a study comparing populations with and without diabetes, Hispanic and Asian individuals exhibited significantly larger FAZ areas than non-Hispanic White participants [12]. Significant differences in retinal and choroidal OCTA parameters were also found between young Black and White subjects [13,14,15] as well as in glaucoma patients of European and African descent [16,17].

Previous studies did, however, not include age- and sex-matched cohorts but rather corrected for confounding factors using statistical approaches. Age–sex matching ensures a more balanced comparison between groups, while statistical adjustment allows for a larger and more diverse sample. Nevertheless, it may not completely eliminate the influence of confounding variables. Understanding ethnic differences between OCTA parameters also has major implications for developing a normative database for clinical application. Several attempts have been made to develop normative databases for OCTA [18,19,20,21], but only one has considered the potential impact of ethnicity [22]. This problem was recently also highlighted for OCT measurements of retinal layers in different ethnic groups [23,24,25,26,27].

The primary objective of this study is to bridge the existing gaps in understanding the quantitative differences in retinal and choroidal OCTA metrics between Caucasian and Asian populations. We included age- and sex-matched cohorts of healthy subjects in two study centers, utilizing identical protocols and OCTA machines to directly compare and identify inter-racial differences in OCTA parameters. By focusing specifically on these two distinct ethnic groups and ensuring methodological consistency, this research aims to provide more precise insights into the unique retinal and choroidal vascular characteristics of Caucasian and Asian individuals.

## 2. Materials and Methods

### 2.1. Study Design

This prospective two-center, cross-sectional study included healthy Chinese and Caucasian participants. It was approved by each individual review board: the Emergency University Hospital Bucharest Institutional Review Board and the Singapore Eye Research Institute Board (ID: 11285, Approval Date: September 2020), and conducted in accordance with the Declaration of Helsinki, in which written informed consent was obtained from participants.

### 2.2. Study Participants

This study recruited 278 healthy participants, of which 145 were Chinese and 133 were Caucasian. All participants were at least 18 years old, provided written informed consent, and underwent a complete ophthalmological examination. The examination included visual acuity (ETDRS charts), ocular refraction, intraocular pressure measurement, anterior segment examination (biomicroscopy and fundoscopy), axial length measurement, OCTA scans of the macula, and visual field testing.

We recruited two groups of healthy controls. The first group comprised Asians from the SIENA and VIBE 3 normal cohorts at the Singapore Eye Research Institute. The second group consisted of healthy Caucasians recruited as controls for a separate multiple sclerosis study conducted at the Emergency University Hospital Bucharest. Participants with any of the following conditions were excluded: namely, glaucoma (clinically diagnosed, suspected, or self-reported), any form of retinopathy, age-related macular degeneration, other clinically significant eye diseases, regular medication use, and severe medical history (as determined by the investigators). The two control groups were age- and gender-matched (1:1). All participant recruitment and examinations occurred between September 2018 and June 2021.

### 2.3. Ophthalmic Examination

All participants underwent a comprehensive ophthalmic examination which included refraction, axial length measurement (IOL Master V3.01, Carl Zeiss Meditec AG, Jena, Germany), visual field testing (Humphrey Field Analyzer 3, Carl Zeiss Meditec, Dublin, CA, USA), and intraocular pressure measurement (Goldmann applanation tonometry). The spherical equivalent was calculated for each participant using the formula spherical power + (cylinder power/2), where all were diopters.

### 2.4. Optical Coherence Tomography Angiography

All participants underwent the macular OCTA scans using the Zeiss Cirrus HD-5000 Spectral-Domain OCT with AngioPlex OCTA (Carl Zeiss Meditec, Dublin, CA, USA). This advanced technology provides high-resolution images (5 µm axial and 15 µm transverse) at a rapid scan speed (68,000 A-scans/second) using a central wavelength of 840 nm. To minimize motion artifacts, technicians employed the FastTrac™ program integrated in Zeiss Cirrus 5000 OCT. Each participant received a 3 × 3 mm^2^ macular scan with isotropic sampling (245 × 245 pixels). Four consecutive B-scans were captured at each location to enable analysis of angiographic information using an optical microangiography protocol [28].

A masked grader then evaluated all OCTA scans for quality. This assessment ensured proper image alignment, segmentation, and signal strength (with a threshold of less than 6 for exclusion). The grader also identified significant motion artifacts, floaters impacting signal, misalignment, or incorrect segmentation [29,30]. Scans with these issues were excluded from the analysis. If both of a participant’s eyes had good-quality scans, one eye was chosen randomly for further analysis. Details on excluded images can be found in Supplementary Materials: Figure S1.

Following the quality assessment of the OCTA scans, we used automated segmentation software (Carl Zeiss Meditec, version 11.0.0.29946) to separate the images into two capillary plexus layers: superficial (SCP) and deep (DCP). These layers correspond to specific retinal structures. The SCP encompasses the inner limiting membrane, nerve fiber layer, ganglion cell layer, and inner plexiform layer, while the DCP includes the inner nuclear layer and outer plexiform layer [31]. Additionally, the choriocapillaris (CC) vasculature, located beneath the retinal pigment epithelium, was extracted for analysis.

The automated instrument software was used to verify the correct segmentation of the images without requiring any additional manual analysis. Special removal software, already integrated into the instrument, was used to resolve projection artifacts from the overlying retinal circulation visible at the DCP level.

**Figure 1:**
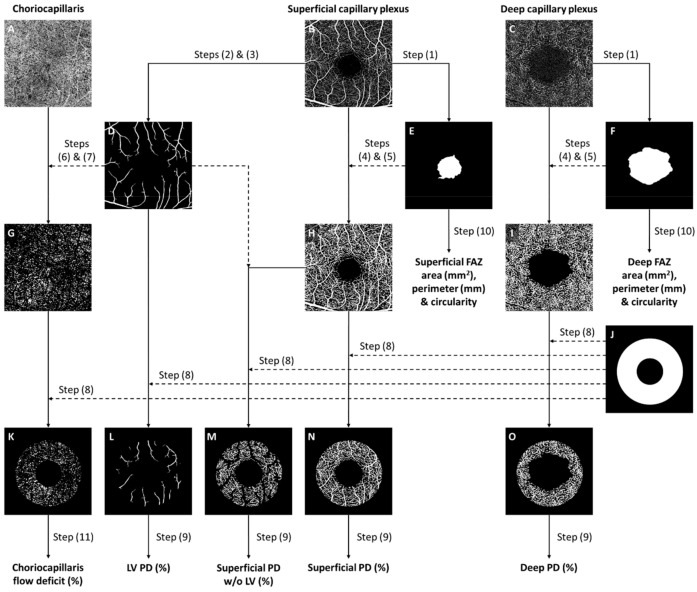
The framework of optical coherence tomography angiography (OCTA) image post-processing. (**A**–**C**) Raw OCTA images extracted from the OCTA machine. (**D**) Large vessels (LVs) segmented and binarized from the superficial capillary plexus. (**E**,**F**) Foveal avascular zones (FAZs) manually delineated from the superficial and deep capillary plexuses. (**G**) Choriocapillaris flow deficits (FDs) binarized from the OCTA image. Large vessel artefacts were masked prior to binarization. (**H**,**I**) Vessels binarized from the superficial and deep capillary plexuses. FAZ regions were masked from the binarized images. (**J**) A magnification-corrected fovea-centered annulus mask with an inner diameter of 1.0 mm and an outer diameter of 2.5 mm. (**K**) Binarized choriocapillaris FDs overlaid with annulus mask to perform regional quantification of FDs. (**L**–**O**) Binarized vascular images overlaid with an annulus mask to perform regional quantification of perfusion density (PD).

Next, the segmented OCTA images (SCP, DCP, and CC) were loaded into a custom MATLAB (The MathWorks Inc., Natick, MA, USA, version R2020b) program for further analysis. The specific processing steps are previously described elsewhere [32,33,34]. The steps are summarized in Figure 1 as follows: (1) manual annotation of the foveal avascular zone (FAZ) of the superficial and deep vascular plexus [35]; (2) enhancement of the contrast of large vessels (LVs) in the SCP by using Gabor- and Hessian-based filters to enhance the contrast of large vessels (LVs) in the SCP; (3–4) binarization of the vessels in the SCP and DCP by thresholding at the mean intensity of the respective images [36,37]; (5) masking of the FAZ regions; (6) removal of large vessel artefacts from the CC slab; (7) binarization of the flow deficits (FDs) in the CC by thresholding 1 standard deviation below the mean intensity of the image; and (8) performing analysis with a fovea-centered annulus that has an inner diameter of 1.0 mm and outer diameter of 2.5 mm.

To correct for ocular magnification, OCT scans were rescaled using Bennett’s formula based on eye length measurements [38], s_actual = p × q × s, where s_actual represents the actual scan length, p represents the magnification factor for the camera of the imaging system, q represents the magnification factor of the eye (q = 0.01306 × (axial length − 1.82)), and s represents the scanning size of the OCTA protocol (9) perfusion density (PD) calculation of the vessels in the retinal layers computed as the percentage of vessel area per total imaged area in the annulus region of measurement and (10) extraction of the FAZ area, perimeter, and circularity (computed as the ratio between the perimeter of the FAZ and the perimeter of an equivalent circle) for both the superficial and deep layer (11) computation of CC FDs as the percentage of flow deficits area per total imaged area in the annulus region.

### 2.5. Statistical Analyses

To evaluate group differences and relationships between ethnicity and OCTA measurements, statistical analyses were performed using StataSE 16.1 software (StataCorp LLC, College Station, TX, USA). Continuous variables were compared between the ethnic groups using independent *t*-tests and chi-square tests to compare categorical variables. The results for continuous variables are presented as mean values accompanied by standard deviation (SD).

Furthermore, multivariable linear regression models were employed to assess how OCTA metrics are associated with ethnicity after accounting for potential confounding factors. These factors included age, diabetes, hypertension, signal strength, spherical equivalent, intraocular pressure, and axial length. A *p* value of less than 0.05 was considered statistically significant throughout the analysis.

## 3. Results

After removing poor-quality scans (n = 42) and performing age- and gender-matching (n = 45), 87 people were excluded from the analysis (Figure S1). Therefore, 191 healthy participants were included in the analysis, 92 of whom were Chinese and 99 of whom were Caucasians.

There was no significant difference in age and gender between the Chinese and Caucasian participants (Table 1). The age of participants was 43 ± 14 years, and 66% were females. Potential confounders such as diabetes, hypertension, and signal strength were also similar in the two study groups (*p* > 0.150). Chinese participants had longer axial length, higher spherical equivalent, and higher intraocular pressure than Caucasian participants (*p* < 0.001). This difference has, however, been accounted for by scaling the annulus region of analysis and further statistical adjustments.

Table 2 presents OCTA metrics in Chinese and Caucasian eyes after adjusting for age, diabetes, hypertension, signal strength, intraocular pressure, and axial length. Chinese eyes demonstrated significantly higher SCP (42.4 ± 2.6% vs. 40.9 ± 2.5%; *p* < 0.001) and a higher SCP without LVs (29.6 ± 2.6% vs. 28.6 ± 2.4%; *p* = 0.002) compared to Caucasians.

We observed a larger superficial FAZ area (0.34 ± 0.1 mm^2^ vs. 0.28 ± 0.1 mm^2^; *p* = 0.004), a wider superficial FAZ perimeter (2.41 ± 0.6 mm vs. 2.14 ± 0.5 mm; *p* = 0.003), more circular deep FAZ (1.08 ± 0.1 vs. 1.11 ± 0.1; *p* = 0.001), and a higher CC FD density (17.2 ± 1.7% vs. 16.7 ± 1.6%; *p* = 0.040) in Chinese participants compared to Caucasians.

No significant differences were observed in the LVs, DCP, superficial FAZ circularity, and deep FAZ area and perimeter, as well as CC FD size and numbers (*p* > 0.079).

**Figure 2:**
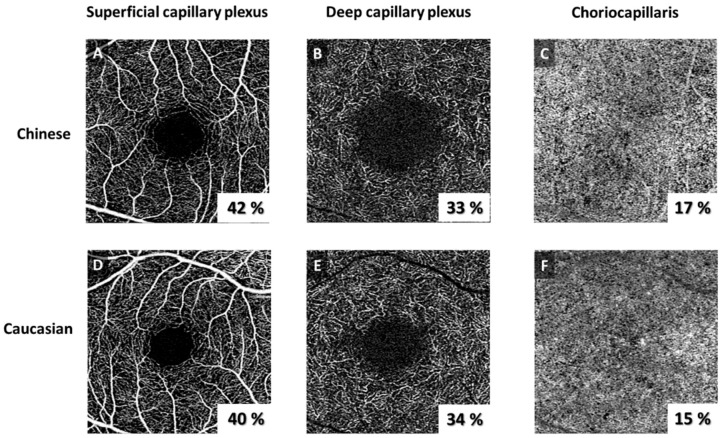
Optical coherence tomography angiography (OCTA) images of a healthy Chinese participant (**A**–**C**) and healthy Caucasian participant (**D**–**F**) of the superficial capillary plexus (SCP; (**A**,**D**)), deep capillary plexus (DCP; (**B**,**E**)), and choriocapillaris (CC; (**C**,**F**)). The eye of the Chinese participant demonstrated significantly higher SCP (42% vs. 40%), a larger superficial FAZ area (0.60 mm^2^ vs. 0.37 mm^2^), wider superficial FAZ perimeter (3.0 mm vs. 2.6 mm), more circular deep FAZ (1.09 vs. 1.11), and higher CC FD density (17% vs. 15%) compared to the Caucasian participant.

The purpose of Figure 2 is to expose two clinic cases, on one hand the Chinese participant and on the other the Caucasian one. The three layers are visible, as a result of the imagery acquired with OCTA: SCP, DCP, and CC. Comparing these images for each individual layer, important differences can be observed, relevant from a statistical point of view and stated previously.

## 4. Discussion

Little is known about the differences in retinal and choroidal microvasculature between ethnicities/races, especially between the Asian and Caucasian populations. As far as we know, this paper is the first to compare these two groups composed of healthy people without ocular pathologies and severe medical conditions. The results suggest significant differences in the retinal microvasculature between healthy Chinese and healthy Caucasian participants. Specifically, Chinese individuals had higher values for some metrics related to the superficial capillary plexus, as well as the choriocapillaris, compared to Caucasians. Additionally, the FAZ was larger in the superficial layer and more circular in the deep layer in the Chinese group than in the Caucasian group. In the case of our study, potential confounding factors such as age, diabetes, hypertension, signal strength, and axial length were also adjusted, indicating that the observed differences were not due to these factors.

Several previous studies have been carried out using OCTA in order to analyze and compare various microvascular variables in different ethnicities. One study showed a significantly lower vascular density in the SCP and DCP in Black subjects as compared to White subjects. At the same time, they observed an enlargement of the FAZ area with a significant decrease in foveolar and parafoveolar vascular density in Black individuals [14]. This is in agreement with another study reporting that there is a decrease in the vascular density of the macular area in Black people compared to White people [13].

Patients with glaucoma of European descent had lower global and sectoral circumpapillary capillary density than patients of African descent [15,16]. A study comparing four groups of different ethnicities, namely non-Hispanic Black, non-Hispanic White, Hispanic, and Asian, also investigated retinal vascular changes using OCTA [12]. In subjects without diabetes, there was no significant difference between groups in terms of vascular perfusion parameters. This is in contrast to the present study, but the sample size was smaller than in our cohort. In patients with mild to moderate non-proliferative diabetic retinopathy, non-Hispanic White people had significantly smaller FAZ areas compared to the other groups. However, there is a general lack of data related to ethnicity-related retinal and choroidal microvascular changes in patients with glaucoma or retinal disease [17].

Overall, our results highlight the importance of understanding the variability in retinal microvasculature between different ethnic groups, as it may have implications for correctly interpreting OCTA results in various retinal diseases. This highlights one of the limitations of the technology in that there is no normative database available for OCTA machines that accounts for different ethnicities [12,13,14,16].

It is currently not known to what degree reduced vascular density may pre-dispose to ocular vascular disease such as diabetic retinopathy. There is evidence that the risk of diabetic retinopathy [39,40,41] differs between Asian and Caucasian populations. Moreover, various studies showed that OCTA metrics are changed in patients with diabetes [10,39,42] and that these alterations can be observed before the onset of diabetic retinopathy [43,44,45]. More specifically, evidence has accumulated that retinal vascular changes in diabetic retinopathy are mainly observed in DCP [37,46,47]. Multiple studies have also shown that FAZ is altered in diabetes in both the SCP and the DCP [48,49,50,51].

Strengths of the study include the recruitment of a well-matched patient cohort, with participants in this study being matched for age and sex. In addition, patients were undergoing similar procedures at both study centers, and common training procedures were scheduled via Internet conferences. In addition, we used a standardized automated way of analyzing the OCTA images that included the removal of projection artefacts as well as the correction of magnification errors. This is important because Asian participants, on average, had longer eye lengths than Caucasians. Whether Littmann’s formula used in the present study truly corrects all the magnification errors due to eye length differences remains unclear.

The limitation of the study includes the relatively small number of participants. In addition, the subjects were studied in two separate locations with different OCTA machines. Although identical machines with the same software release were used, we cannot exclude that there may be systematic differences between the two systems. The subjects included were relatively young and, as such, do not represent the age group that is most likely developing neurodegenerative eye disease.

Since OCTA measurements can vary based on ethnicity, understanding these variations is essential for interpreting the results accurately in diagnosing and monitoring retinal diseases. Unfortunately, current OCTA normative databases do not account for ethnicity. This is a significant limitation, particularly when using the OCTA device for eye disease monitoring. Therefore, factoring in ethnicity alongside OCTA findings could be crucial for the early detection and risk assessment of retinal diseases in different populations.

## 5. Conclusions

A comparative analysis of retinal microvasculature between healthy Chinese and Caucasian subjects, performed using OCTA as a non-invasive tool, revealed significant microvascular differences, particularly in the SCP and FAZ. Longitudinal studies are warranted to investigate whether lower perfusion density is a risk factor for developing ocular vascular diseases. Furthermore, considering ethnicity alongside age and sex when matching cohorts in future case–control studies involving healthy patients and patients with the disease is crucial for the accurate interpretation of OCTA findings. This study highlights the importance of ethnicity in developing normative databases for OCTA analysis, alongside established factors like age and gender.

## Figures and Tables

**Table 1:** Characteristics of participants by ethnicity.

Characteristics	Chinese (*n* = 92)	Caucasian (*n* = 99)	*p* Value *
Age	44 ± 13	42 ± 14	0.311
Gender, female	63 (68%)	63 (64%)	0.480
Diabetes, no	92 (100%)	97 (98%)	0.171
Hypertension, no	75 (82%)	88 (89%)	0.150
Axial length, mm	24.7 ± 1.5	23.2 ± 0.9	**<0.001**
Spherical equivalent, diopters	−2.4 ± 3.3	−0.1 ± 1.9	**<0.001**
Intraocular pressure, mmHg	16.8 ± 3.3	15.1 ± 2.4	**<0.001**
Signal strength, out of 10	9.3 ± 0.9	9.4 ± 1.0	0.682

Data presented are mean (SD) or number (%), as appropriate. * *p* value was obtained with independent *t*-test for continuous variables and chi-square test for categorical variables. **Bold** values denote statistical significance at the *p* < 0.05 level.

**Table 2:** Analysis of optical coherence tomography angiography parameters between ethnicities.

OCTA Metrics	Chinese	Caucasian	
	(*n* = 92)	(*n* = 99)	
	Mean ± SD	Mean ± SD	*p* Value *
Perfusion density (%)			
LVs	6.7 ± 0.8	6.6 ± 0.8	0.729
SCP	42.4 ± 2.6	40.9 ± 2.5	**<0.001**
SCP w/o LVs	29.6 ± 2.6	28.6 ± 2.4	**0.002**
DCP	39.1 ± 3.6	40.1 ± 3.4	0.079
Foveal avascular zone at superficial layer			
Area (mm^2^)	0.34 ± 0.1	0.28 ± 0.1	**0.004**
Perimeter (mm)	2.41 ± 0.6	2.14 ± 0.5	**0.003**
Circularity	1.19 ± 0.2	1.18 ± 0.1	0.599
Foveal avascular zone at deep layer			
Area (mm^2^)	1.14 ± 0.3	1.11 ± 0.3	0.521
Perimeter (mm)	4.04 ± 0.6	4.12 ± 0.6	0.414
Circularity	1.08 ± 0.1	1.11 ± 0.1	**0.001**
Flow deficit in choriocapillaris			
FD density (%)	17.2 ± 1.7	16.7 ± 1.6	**0.040**
FD size (µm^2^)	508 ± 96	496 ± 91	0.466
FD number	1379 ± 129	1407 ± 121	0.163

DCP deep capillary plexus, FD flow deficit, LVs large vessels, OCTA optical coherence tomography angiography, SCP superficial capillary plexus, SD standard deviation. * *p* value was obtained with multivariable linear regression analysis, adjusted for age, diabetes, hypertension, signal strength, spherical equivalent, intraocular pressure, and axial length.

## Data Availability

The datasets used and/or analyzed during the current study are available from the corresponding authors upon reasonable request. The dataset(s) supporting the conclusions of this article is(are) included within the article.

## Supplementary Materials

The following supporting information can be downloaded at: https://www.mdpi.com/article/10.3390/jpm14080834/s1, Figure S1: Participants included in the present study.

## References

[1] Ikram M.K., Ong Y.T., Cheung C.Y., Wong T.Y. (2013). Retinal Vascular Caliber Measurements: Clinical Significance, Current Knowledge and Future Perspectives. Ophthalmologica.

[2] Heitmar R., Blann A.D., Cubbidge R.P., Lip G.Y.H., Gherghel D. (2010). Continuous Retinal Vessel Diameter Measurements: The Future in Retinal Vessel Assessment?. Investig. Opthalmol. Vis. Sci..

[3] Garhofer G., Bek T., Boehm A.G., Gherghel D., Grunwald J., Jeppesen P., Kergoat H., Kotliar K., Lanzl I., Lovasik J.V. (2010). Use of the retinal vessel analyzer in ocular blood flow research. Acta Ophthalmol..

[4] Li X., Wong W.L., Cheung C.Y., Cheng C.-Y., Ikram M.K., Li J., Chia K.S., Wong T.Y. (2013). Racial Differences in Retinal Vessel Geometric Characteristics: A Multiethnic Study in Healthy Asians. Investig. Opthalmol. Vis. Sci..

[5] Rochtchina E., Wang J.J., Taylor B., Wong T.Y., Mitchell P. (2008). Ethnic Variability in Retinal Vessel Caliber: A Potential Source of Measurement Error from Ocular Pigmentation?—The Sydney Childhood Eye Study. Investig. Opthalmol. Vis. Sci..

[6] Smith W., Kotliar K.E., Lammertyn L., Ramoshaba N.E., Vilser W., Huisman H.W., Schutte A.E. (2020). Retinal vessel caliber and caliber responses in true normotensive black and white adults: The African-PREDICT study. Microvasc. Res..

[7] Huang D., Swanson E.A., Lin C.P., Schuman J.S., Stinson W.G., Chang W., Hee M.R., Flotte T., Gregory K., Puliafito C.A. (1991). Optical Coherence Tomography. Science.

[8] Spaide R.F., Fujimoto J.G., Waheed N.K., Sadda S.R., Staurenghi G. (2018). Optical coherence tomography angiography. Prog. Retin. Eye Res..

[9] Ang M., Tan A.C.S., Cheung C.M.G., Keane P.A., Dolz-Marco R., Sng C.C.A., Schmetterer L. (2018). Optical coherence tomography angiography: A review of current and future clinical applications. Graefe’s Arch. Clin. Exp. Ophthalmol..

[10] Chua J., Sim R., Tan B., Wong D., Yao X., Liu X., Ting D.S.W., Schmidl D., Ang M., Garhöfer G. (2020). Optical Coherence Tomography Angiography in Diabetes and Diabetic Retinopathy. J. Clin. Med..

[11] Chua J., Tan B., Ang M., Nongpiur M.E., Tan A.C., Najjar R.P., Milea D., Schmetterer L. (2019). Future clinical applicability of optical coherence tomography angiography. Clin. Exp. Optom..

[12] Laotaweerungsawat S., Psaras C., Haq Z., Liu X., Stewart J.M. (2021). Racial and ethnic differences in foveal avascular zone in diabetic and nondiabetic eyes revealed by optical coherence tomography angiography. PLoS ONE.

[13] Chun L.Y., Silas M.R., Dimitroyannis R.C., Ho K., Skondra D. (2019). Differences in macular capillary parameters between healthy black and white subjects with Optical Coherence Tomography Angiography (OCTA). PLoS ONE.

[14] Massamba N., Mackin A.G., Chun L.Y., Rodriguez S., Dimitroyannis R.C., Bodaghi B., Hariprasad S.M., Skondra D. (2021). Evaluation of flow of chorioretinal capillaries in healthy black and white subjects using optical coherence tomography angiography. Sci. Rep..

[15] Moir J., Rodriguez S.H., Chun L.Y., Massamba N., Skondra D. (2023). Racial differences in quantitative optical coherence tomography angiography findings between older non-diabetics with co-morbidities. PLoS ONE.

[16] Moghimi S., Zangwill L.M., Hou H., Wong B., Proudfoot J., Penteado R.C., Ekici E., Bowd C., Weinreb R.N. (2021). Comparison of Peripapillary Capillary Density in Glaucoma Patients of African and European Descent. Ophthalmol. Glaucoma.

[17] Siesky B., Harris A., Vercellin A.C.V., Guidoboni G., Tsai J.C. (2021). Ocular blood flow as it relates to race and disease on glaucoma. Adv. Ophthalmol. Optom..

[18] Tan B., Sim Y.C., Chua J., Yusufi D., Wong D., Yow A.P., Chin C., Tan A.C.S., Sng C.C.A., Agrawal R. (2021). Developing a normative database for retinal perfusion using optical coherence tomography angiography. Biomed. Opt. Express.

[19] Fernández-Vigo J.I., Kudsieh B., Shi H., Arriola-Villalobos P., Donate-López J., García-Feijóo J., Ruiz-Moreno J.M., Fernández-Vigo J.Á. (2020). Normative database and determinants of macular vessel density measured by optical coherence tomography angiography. Clin. Exp. Ophthalmol..

[20] Fernández-Vigo J.I., Kudsieh B., Shi H., De-Pablo-Gómez-de-Liaño L., Serrano-Garcia I., Ruiz-Moreno J.M., Martínez-de-la-Casa J.M., García-Feijóo J., Fernández-Vigo J.Á. (2020). Normative Database of Peripapillary Vessel Density Measured by Optical Coherence Tomography Angiography and Correlation Study. Curr. Eye Res..

[21] Coscas F., Sellam A., Glacet-Bernard A., Jung C., Goudot M., Miere A., Souied E.H. (2016). Normative Data for Vascular Density in Superficial and Deep Capillary Plexuses of Healthy Adults Assessed by Optical Coherence Tomography Angiography. Invest. Ophthalmol. Vis. Sci..

[22] Munsell M.K., Garg I., Duich M., Zeng R., Baldwin G., Wescott H.E., Koch T., Wang K.L., Patel N.A., Miller J.B. (2023). A normative database of wide-field swept-source optical coherence tomography angiography quantitative metrics in a large cohort of healthy adults. Graefe’s Arch. Clin. Exp. Ophthalmol..

[23] Ho H., Tham Y.-C., Chee M.L., Shi Y., Tan N.Y.Q., Wong K.-H., Majithia S., Cheung C.Y., Aung T., Wong T.Y. (2019). Retinal Nerve Fiber Layer Thickness in a Multiethnic Normal Asian Population: The Singapore Epidemiology of Eye Diseases Study. Ophthalmology.

[24] Nousome D., Mckean-Cowdin R., Richter G.M., Burkemper B., Torres M., Varma R., Jiang X. (2021). Retinal Nerve Fiber Layer Thickness in Healthy Eyes of Black, Chinese, and Latino Americans: A Population-Based Multiethnic Study. Ophthalmology.

[25] Perez C.I., Chansangpetch S., Mora M., Nguyen A., Zhao J., Han Y., Lin S.C. (2021). Ethnicity-Specific Database Improves the Diagnostic Ability of Peripapillary Retinal Nerve Fiber Layer Thickness to Detect Glaucoma. Am. J. Ophthalmol..

[26] Chua J., Schwarzhans F., Nguyen D.Q., Tham Y.C., Sia J.T., Lim C., Mathijia S., Cheung C., Tin A., Fischer G. (2020). Compensation of retinal nerve fibre layer thickness as assessed using optical coherence tomography based on anatomical confounders. Br. J. Ophthalmol..

[27] Chua J., Schwarzhans F., Wong D., Li C., Husain R., Crowston J.G., Perera S.A., Sng C.C.A., Nongpiur M.E., Majithia S. (2022). Multivariate Normative Comparison, a Novel Method for Improved Use of Retinal Nerve Fiber Layer Thickness to Detect Early Glaucoma. Ophthalmol. Glaucoma.

[28] Wang R.K., An L., Francis P., Wilson D.J. (2010). Depth-resolved imaging of capillary networks in retina and choroid using ultrahigh sensitive optical microangiography. Opt. Lett..

[29] Chua J., Chin C.W.L., Hong J., Chee M.L., Le T.-T., Ting D.S.W., Wong T.Y., Schmetterer L. (2019). Impact of hypertension on retinal capillary microvasculature using optical coherence tomographic angiography. J. Hypertens..

[30] Chua J., Chin C.W.L., Tan B., Wong S.H., Devarajan K., Le T.-T., Ang M., Wong T.Y., Schmetterer L. (2019). Impact of systemic vascular risk factors on the choriocapillaris using optical coherence tomography angiography in patients with systemic hypertension. Sci. Rep..

[31] Rosenfeld P.J., Durbin M.K., Roisman L., Zheng F., Miller A., Robbins G., Schaal K.B., Gregori G. (2016). ZEISS AngioplexTM Spectral Domain Optical Coherence Tomography Angiography: Technical Aspects. OCT Angiography in Retinal and Macular Diseases.

[32] Chua J., Le T., Sim Y.C., Chye H.Y., Tan B., Yao X., Wong D., Ang B.W.Y., Toh D., Lim H. (2022). Relationship of Quantitative Retinal Capillary Network and Myocardial Remodeling in Systemic Hypertension. J. Am. Heart Assoc..

[33] Chua J., Hu Q., Ke M., Tan B., Hong J., Yao X., Hilal S., Venketasubramanian N., Garhöfer G., Cheung C.Y. (2020). Retinal microvasculature dysfunction is associated with Alzheimer’s disease and mild cognitive impairment. Alzheimer’s Res. Ther..

[34] Tan B., Sim R., Chua J., Wong D.W.K., Yao X., Garhöfer G., Schmidl D., Werkmeister R.M., Schmetterer L. (2020). Approaches to quantify optical coherence tomography angiography metrics. Ann. Transl. Med..

[35] Shahlaee A., Pefkianaki M., Hsu J., Ho A.C. (2016). Measurement of Foveal Avascular Zone Dimensions and its Reliability in Healthy Eyes Using Optical Coherence Tomography Angiography. Am. J. Ophthalmol..

[36] Frangi A.F., Niessen W.J., Vincken K.L., Viergever M.A. Multiscale vessel enhancement filtering. Proceedings of the Medical Image Computing and Computer-Assisted Interventation—MICCAI’98.

[37] Tan B., Chua J., Lin E., Cheng J., Gan A., Yao X., Wong D.W.K., Sabanayagam C., Wong D., Chan C.M. (2020). Quantitative Microvascular Analysis with Wide-Field Optical Coherence Tomography Angiography in Eyes with Diabetic Retinopathy. JAMA Netw. Open.

[38] Bennett A.G., Rudnicka A.R., Edgar D.F. (1994). Improvements on Littmann’s method of determining the size of retinal features by fundus photography. Graefe’s Arch. Clin. Exp. Ophthalmol..

[39] Gupta R., Misra A. (2016). Epidemiology of microvascular complications of diabetes in South Asians and comparison with other ethnicities. J. Diabetes.

[40] Raymond N.T., Varadhan L., Reynold D.R., Bush K., Sankaranarayanan S., Bellary S., Barnett A.H., Kumar S., O’Hare J.P. (2009). Higher Prevalence of Retinopathy in Diabetic Patients of South Asian Ethnicity Compared with White Europeans in the Community. Diabetes Care.

[41] Pardhan S., Gilchrist J., Mahomed I. (2004). Impact of age and duration on sight–threatening retinopathy in South Asians and Caucasians attending a diabetic clinic. Eye.

[42] Johannesen S.K., Viken J.N., Vergmann A.S., Grauslund J. (2019). Optical coherence tomography angiography and microvascular changes in diabetic retinopathy: A systematic review. Acta Ophthalmol..

[43] Furino C., Montrone G., Cicinelli M.V., Balestra S., Grassi M.O., Reibaldi M., Boscia F., Alessio G. (2020). Optical coherence tomography angiography in diabetic patients without diabetic retinopathy. Eur. J. Ophthalmol..

[44] Zeng Y., Cao D., Yu H., Yang D., Zhuang X., Hu Y., Li J., Yang J., Wu Q., Liu B. (2019). Early retinal neurovascular impairment in patients with diabetes without clinically detectable retinopathy. Br. J. Ophthalmol..

[45] Rosen R.B., Andrade Romo J.S., Krawitz B.D., Mo S., Fawzi A.A., Linderman R.E., Carroll J., Pinhas A., Chui T.Y.P. (2019). Earliest Evidence of Preclinical Diabetic Retinopathy Revealed Using Optical Coherence Tomography Angiography Perfused Capillary Density. Am. J. Ophthalmol..

[46] Tan B., Lim N.-A., Tan R., Gan A.T.L., Chua J., Nusinovici S., Cheung C.M.G., Chakravarthy U., Wong T.Y., Schmetterer L. (2022). Combining retinal and choroidal microvascular metrics improves discriminative power for diabetic retinopathy. Br. J. Ophthalmol..

[47] Kaizu Y., Nakao S., Arima M., Wada I., Yamaguchi M., Sekiryu H., Hayami T., Ishikawa K., Ikeda Y., Sonoda K. (2019). Capillary dropout is dominant in deep capillary plexus in early diabetic retinopathy in optical coherence tomography angiography. Acta Ophthalmol..

[48] Aitchison R.T., Kennedy G.J., Shu X., Mansfield D.C., Kir R., Hui J., Shahani U. (2022). Measuring the foveal avascular zone in diabetes: A study using optical coherence tomography angiography. J. Diabetes Investig..

[49] Oliverio G.W., Ceravolo I., Bhatti A., Trombetta C.J. (2021). Foveal avascular zone analysis by optical coherence tomography angiography in patients with type 1 and 2 diabetes and without clinical signs of diabetic retinopathy. Int. Ophthalmol..

[50] Onoe H., Kitagawa Y., Shimada H., Shinojima A., Aoki M., Urakami T. (2020). Foveal avascular zone area analysis in juvenile-onset type 1 diabetes using optical coherence tomography angiography. Jpn. J. Ophthalmol..

[51] Di G., Weihong Y., Xiao Z., Zhikun Y., Xuan Z., Yi Q., Fangtian D. (2016). A morphological study of the foveal avascular zone in patients with diabetes mellitus using optical coherence tomography angiography. Graefe’s Arch. Clin. Exp. Ophthalmol..

