# Supplementary material for: Comparing Optical Coherence Tomography Angiography Metrics in Healthy Chinese and Caucasian Adults
Source: J Pers Med. 2024 Aug 6;14(8):834. doi: 10.3390/jpm14080834 (PMC11355270; doi:10.3390/jpm14080834)
Supplement: Supplementary file 1 [file jpm-14-00834-s001.zip › jpm-3057131-supplementary.pdf]

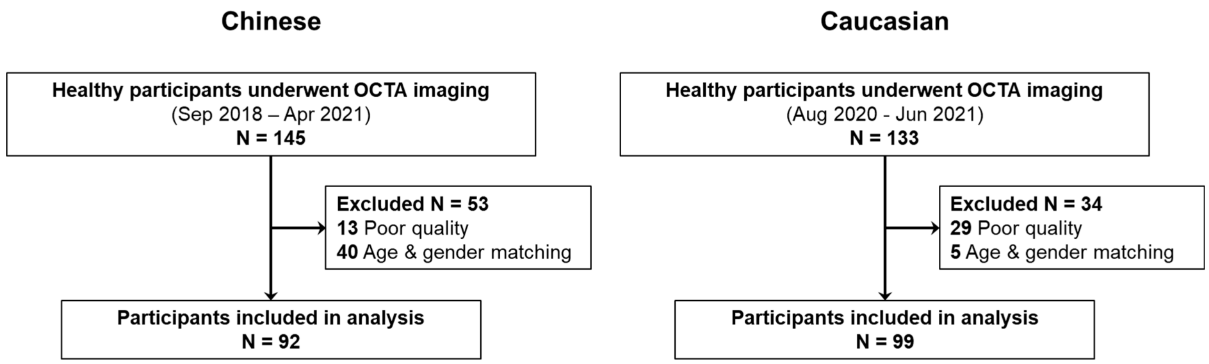

Figure S1 Participants included in the present study. Of the 278 participants, we excluded 42 participants due to poor scan quality, and further excluded 45 participants upon matching of age and gender between the Chinese and Caucasian cohorts. Among the 191 healthy participants included for the analysis, 92 were Chinese participants, and 99 were Caucasian participants.
